# Sleep dependent autonomic correlates of affect and arousal in younger and older adults

**DOI:** 10.1093/sleepadvances/zpag083

**Published:** 2026-07-28

**Authors:** Anjana Subramoniam, Pin-Chun Chen, Negin Sattari, Sara C Mednick, Lauren N Whitehurst

**Affiliations:** Department of Psychiatry, College of Medicine, University of Pittsburgh, Pittsburgh, Pennsylvania, United States; Department of Psychology, Royal Holloway, University of London, Egham, TW20 0EX, United Kingdom; Cognitive Science School of Social Sciences, University of California, Irvine, California, United States; Cognitive Science School of Social Sciences, University of California, Irvine, California, United States; Department of Psychology, Yale University, New Haven, Connecticut, United States; Department of Black Studies, Yale University, New Haven, Connecticut, United States; Wu Tsai Institute, Yale University, New Haven, Connecticut, United States

**Keywords:** heart rate variability, autonomic nervous system, emotion, aging, sleep

## Abstract

**Study Objectives:**

Aging alters affect, with older vs. younger adults exhibiting greater positive and reduced negative affect. Sleep and autonomic factors contribute to this change, yet it is untested whether sleep-dependent autonomic activity plays a role. The current study examined bidirectional associations between affect, arousal, sleep architecture, and cardiac autonomic activity across a daytime nap in healthy younger and older adults.

**Materials and Methods:**

Polysomnography and electrocardiogram were collected during a midday nap. Sleep architecture and autonomic activity outputs (i.e. high frequency, HF-HRV, and low frequency, LF-HRV, heart rate variability) were extracted. Affect ratings (assessed *via* the Multiple Affect Adjective Checklist), captured before and after the nap, were considered along two continua: (1) positive and negative valence and (2) low and high arousal. Positivity ratings (positive – negative valence) were determined within the two arousal categories.

**Results:**

Younger adults reported stronger positivity for low- relative to high-arousal affect after the nap, whereas positivity did not differ by arousal in older adults. Wake and NREM autonomic activity was lower in older than younger adults. Higher waking and NREM LF activity were associated with stronger post-nap positivity, whereas higher HF activity was associated with diminished post-nap positivity. Pre-sleep positivity ratings were not meaningfully associated with HRV during sleep. Sleep and wake findings were broadly consistent across age cohorts.

**Conclusion:**

The current study extends previous work linking sleep-related autonomic activity and psychosocial outcomes and offers that autonomic activity during wake and sleep may work synergistically to calibrate affective experiences across the lifespan.

## Graphical Abstract

**Graphical Abstract:**
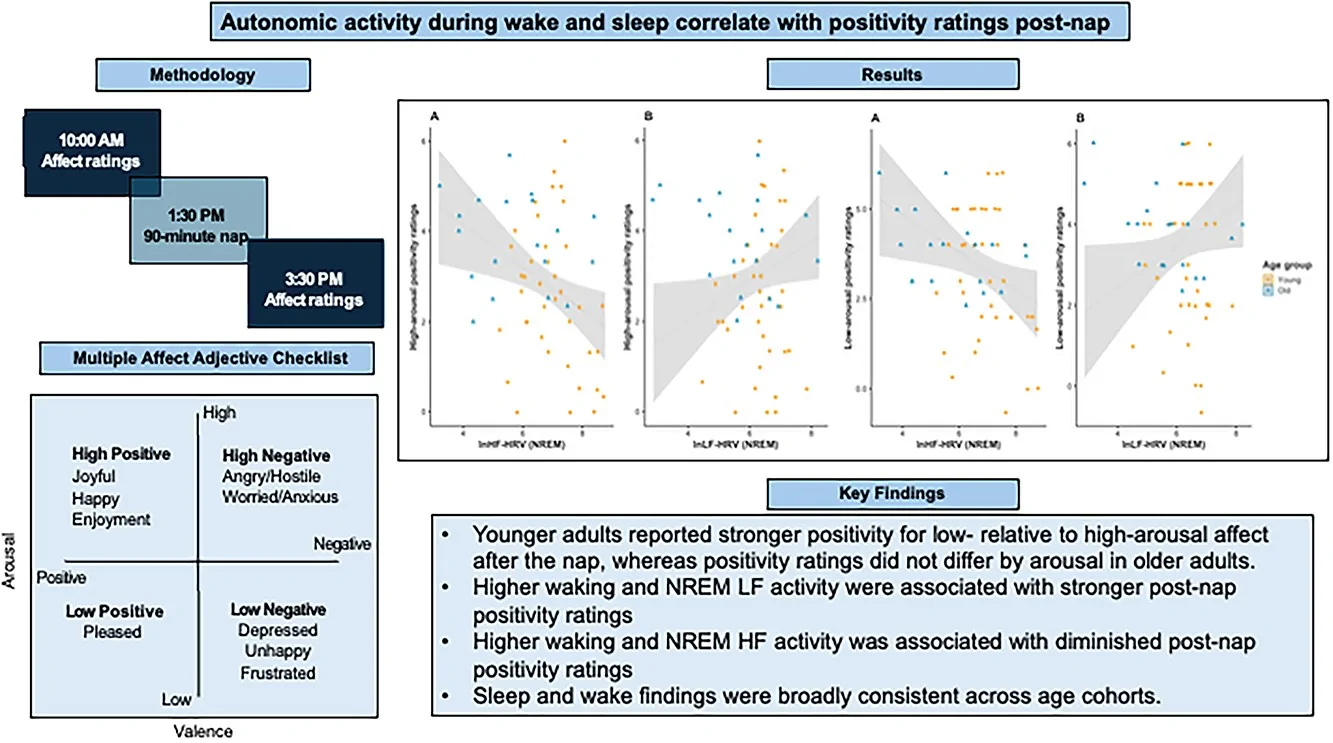

**Statement of Significance**

Empirical studies in sleep and affective science have focused primarily on neural mechanisms, overlooking the autonomic processes that unfold during sleep, a critical period for emotional regulation. This study investigated whether autonomic activity, during wake and sleep, is associated with affect ratings, pre- and post-sleep, in an older and younger adult cohort. Pre-sleep affect was not reliably associated with wake or sleep autonomic activity. Yet, resting and sleep-specific autonomic activity were reliably linked to post-sleep affect revealing that, for healthy adults, autonomic activity across wake and sleep likely conspire to balance affective experiences.

## Introduction

Aging can modulate affective experience—an individual's emotional state or mood. Positive affect encompasses emotions such as joy, enthusiasm, and contentment, while negative affect implicates emotions such as sadness, anxiety, or frustration [1]. Cross-sectional studies have demonstrated that older adults report greater positive affect and reduced negative affect when compared to younger adults [2–5]. For example, memory studies indicate that older adults tend to recall more positive than negative information over several retention delay intervals [6–9]. Increased positive affect with aging is also coupled with a reduction in negative affective states like anger, anxiety, fear, and sadness [10–16]. This tendency to preferentially attend to, retain, and recall positive over negative information is commonly referred to as the age-related “positivity effect” or “positivity bias” [8, 17]. The positivity effect is often measured using self-reported surveys assessing two dimensions of an affective state: (1) valence, the intrinsic positivity or negativity of emotion, and (2) arousal, the intensity or activation level of the emotional response [18], with prevalent responses to positive valence, and dampened responses to high arousal information serving as core driving features of the age-related positivity effect [19]. Although psychosocial motivations for the positivity effect with aging have been hypothesized [4, 20–23], the physiological underpinnings that contribute to these age-dependent affective patterns require further investigation.

Quality sleep is a driver of healthy affective regulation [24–26]. For example, adequate nighttime sleep is associated with increased positive affect [27, 28], while sleep disruptions are correlated with increased negative and decreased positive affect (see reviews [29, 30]). Insufficient amounts of non-rapid eye movement (NREM) and REM sleep are linked to heightened negative reactivity [31–33] as well as diminished positive affect in response to positive events [34, 35]. Beyond sleep stage durations, specific NREM features also appear to play a role in affective regulation. Greater power in the delta band (0.5–4 Hz) is correlated with higher positive affect after sleep in older adults [36], and higher sigma activity (11–16 Hz) predicts more positive relative to negative emotions following a nap [37]. Importantly, experiencing negative emotions prior to sleep can also induce sleep disruptions [38], indicating sleep and affect share a bidirectional relationship. Sleep difficulties progress with age, as sleep efficiency and continuity decrease [39]. Age-dependent changes in electrophysiological events during sleep are apparent, including reductions in NREM minutes, delta activity, and sigma activity [39–45], suggesting that decreased NREM sleep may be a probable factor related to affectivity shifts with aging.

The autonomic nervous system (ANS) may also account for age-related shifts in affective processing. The sympathetic and parasympathetic branches of the ANS dynamically regulate physiological responses such as heart rate, blood pressure, respiration, and hormones in response to affective states (see reviews [46–48]). Changes in the sympathetic, associated with energy mobilization, and the parasympathetic, which orchestrates restorative functions of the ANS, have been used to characterize affective responses. Heart rate variability (HRV), a noninvasive technique that assesses interbeat heart intervals, reflects the dynamic interplay between the sympathetic and parasympathetic branches of the ANS and can provide insight into cardiac autonomic regulation, and its association with affective experience (see reviews [49–51]). For example, RMSSD (time-domain based measure of HRV reflecting parasympathetic activity) during wake has been correlated with affective self-regulation and flexibility [52], emotional resilience [53], improved affective cognition [54], and a lower risk of affective psychopathology [55]. High frequency (HF) HRV—primarily reflecting parasympathetic outflow [56, 57] correlates with improved emotional well-being [55, 58, 59] and decreased anxiety [60, 61], primarily in young adults. Further, higher parasympathetically mediated HRV during wake, is linked to increased positive emotions across the day [59]. However, some studies reporting null associations between resting HRV and affective outcomes suggest that links between affective state and HRV may be more context-dependent than previously recognized [62]; highlighting the need for further research to clarify when and how the ANS contributes to affective states.

In young healthy adults, at sleep onset, total cardiac autonomic activity decreases and shifts toward parasympathetic dominance [63, 64]. Importantly, parasympathetic dominance during sleep substantially diminishes with aging. Compared with younger adults, older adults exhibit reduced vagally mediated HRV, reflected in lower HF power and RMSSD, particularly during N2 and N3 sleep [65]. Yet, it is hypothesized that positive affect, including experiencing joy, gratitude, and social connectedness, may blunt autonomic declines with aging and support ANS functioning in older adults [66]. While prior research has linked the positivity effect and ANS, these studies have focused mostly on ANS activity during wakefulness. This focus overlooks fluctuations in autonomic activity during sleep [63, 64, 67, 68], as well as age related variability in sleep-dependent ANS activity, [65] which is the focus of this investigation.

The current study examined associations between affect ratings (i.e. positive and negative affect, and low and high arousal affect) and neural and cardiac autonomic activity across a daytime nap in healthy younger and older adults. Using a cross-sectional design, we investigated two related questions. First, we examined whether sleep-linked cardiac indicators together with NREM neural sleep features, were associated with post sleep positive and negative affect and arousal following a nap. Second, we tested whether affect prior to sleep was associated with autonomic activity during wake and NREM sleep. In both probes, we assessed whether autonomic associations with affect and arousal varied based on age cohort. Given that sleep-related affective disturbances are observed in both older and younger adults [29, 30, 69, 70] we expected that similar sleep mechanisms may be linked to affect across age cohorts. In particular, age related changes in autonomic activity during sleep may partially account for the age-related positivity effect. We tested the following set of hypotheses: (1) Older, relative to younger adults, would report more positive compared to negative affective rating’s and more low compared to high arousal ratings; (2) Older adults would have dampened parasympathetically mediated autonomic activity during sleep; (3) Duration in NREM sleep, hallmark features of NREM sleep (i.e. delta and sigma power), and parasympathetically mediated autonomic activity during NREM sleep would be associated with positivity ratings after sleep; (4) NREM sleep and autonomic associations would be most pronounced in younger adults and lastly, to determine if these relationships were bidirectional we assessed associations between affective states prior to the nap and autonomic features during a resting wake episode and during the nap and expected that (5) exhibiting higher levels of positive affect prior to the nap would result in higher HF-HRV during the nap.

## Materials and Methods

### Participants

The University of California, Riverside Human Research Review Board approved all testing procedures. Young adults [N = 59, Age (Mean ± SD = 20.23 ± 2.62), 24 males] and older adults [N = 52, Age (Mean ± SD = 68.62 ± 5.55), 20 males] recruited from a community sample, participated in the study. The demographics associated with this sample are displayed in Table 1. Participants were selected based on having no personal history of neurological, psychological, cardiovascular, psychiatric, sleep disorders, or other chronic illnesses. This was determined during telephone and in-person research interviews. Informed consent was obtained from the participants during an in-person orientation. All participants reported a regular sleep–wake schedule, defined as 6–9 hours/night. The participants received financial compensation for their contribution.

**Table 1:** Sample characteristics for younger and older adult participants

	Younger M (SD)	Older M (SD)
	N = 59	N = 52
Age	20.24 (2.62)	68.62(5.55)
Male/Female	24/35	18/34
Ethnicity		
Asian	32	11
African American	1	3
Caucasian	6	26
Hispanic	19	6
Others (unspecified)	1	6
BMI^*^	24.33(4.99)	28.27(5.29)
ESS^*^	7.19(3.58)	6.61(3.10)

**Note.** Values are presented as mean (SD) for continuous variables and n for categorical variables. BMI = body mass index; ESS = Epworth Sleepiness Scale.

### Study procedure

One week before the nap visit, participants wore a wrist-based activity monitor/actigraph (ActiWatch Spectrum, Philips Respironics, USA) for screening/compliance purposes and completed electronic sleep diaries to ensure they received an average of 6–9 hours of sleep each night. No actigraphy measures were used in any of the reported analyses. Caffeine and alcohol were not permitted 24 hours before and throughout the nap visit. Participants were instructed to maintain their regular sleep schedule prior to the study visit. No preparatory sleep restriction or sleep deprivation procedures were implemented before the in-lab visit. On the visit day, participants arrived at the lab at 10 a.m. and completed a modified version of the Multiple Affect Adjective Checklist (MAACL-R) [71]. After completing this questionnaire, polysomnography electrodes were attached to participants. Following electrode placement, participants were required to lay in a supine position for 5-minutes which was used to calculate waking cardiac activity. After the 5-min resting period, participants were allowed a 2-hour opportunity to achieve up to 90-minutes of sleep. Participants were woken up if they achieved 90 minutes of sleep time. Shortly after the nap episode, participants were asked to complete the MAACL-R.

### Measures

#### Affective state

**Figure 1:**
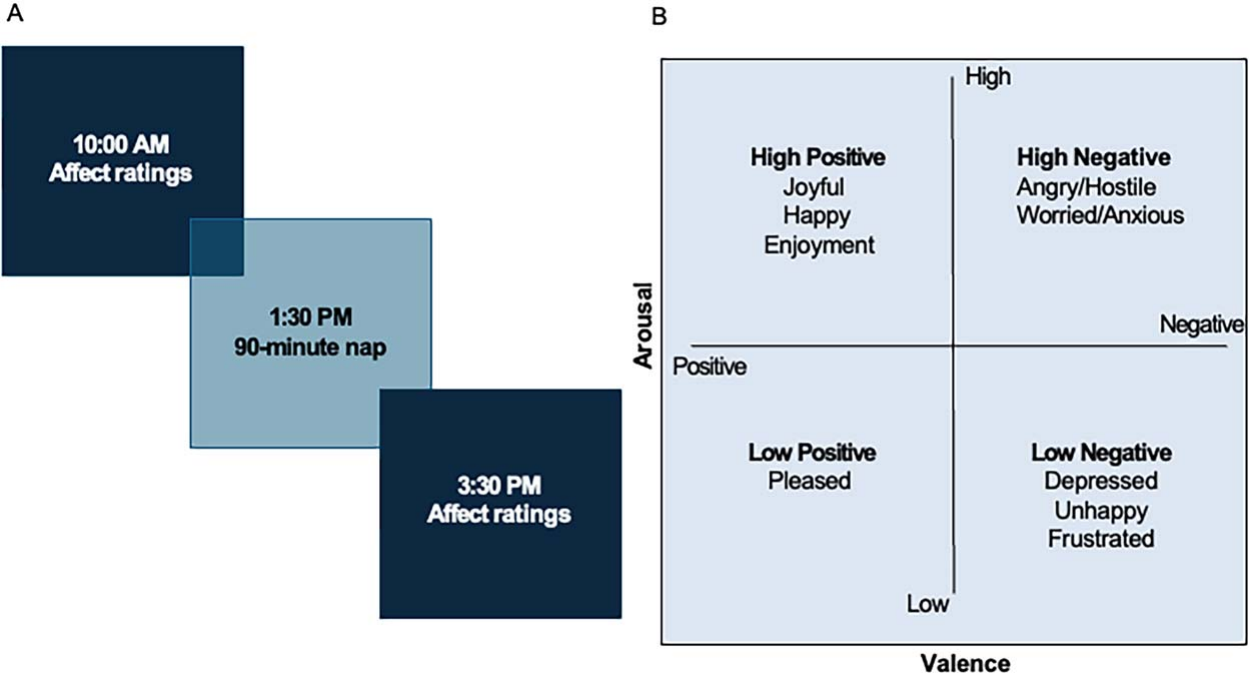
(A) Experimental timeline. Participants completed baseline emotional ratings at 10:00 a.m., followed by a 90-minute nap opportunity at 1:30 p.m. post-nap emotional ratings were obtained at 3:30 p.m. to assess changes in affect following sleep. (**1B**) **Arousal–valence affect categories**. Emotional ratings were organized into four categories based on arousal (low to high) and valence (positive to negative): High-arousal positive (joyful, happy), low-arousal positive (pleased), high-arousal negative (angry, anxious), and low-arousal negative (depressed, unhappy, frustrated).

The Multiple Affect Adjective Checklist (MAACL-R) assesses positive and negative emotions. The checklist includes nine prompts: happy, pleased, joyful, enjoyment/fun, angry/hostile, worried/anxious, frustrated, depressed/blue, and unhappy. On a scale of 1 = not at all to 7 = extremely, participants were asked to rate how much they were currently experiencing each emotional state (Refer Figure 1). To assess valence and arousal components of emotional experience modulated by sleep, we parsed these emotional states into (1) low arousal/positive emotional states (pleased), (2) high arousal/positive emotional states (happy, joyful, and enjoyment), (3) low arousal/negative emotional states (depressed, frustrated, and unhappy), and (4) high arousal/negative emotional states (angry/hostile, worried/anxious). For each category, an average score was computed by summing ratings across items within the category and dividing by the number of items. We refer to these category averages as affect ratings. Refer to Supplemental Figure S1. From these category scores, we derived positivity ratings, calculated as positive minus negative ratings within high and low arousal. To facilitate interpretation of the results, we adopt a consistent terminological convention: *affect ratings* denotes the raw MAACL summary scores, while *positivity ratings* are used exclusively for the derived difference scores. Positivity ratings represent the difference between positive and negative affect ratings within each arousal level (low vs. high), with higher values indicating greater positive than negative endorsed affect endorsed. Positivity ratings were only entered into the subsequent statistical analyses, and this terminological distinction is preserved throughout the Results.

#### Sleep recording and scoring

EEG data was acquired using a 32-channel cap (EASYCAP GmbH) with Ag/AgCI electrodes positioned following the global 10–20 System. Active scalp recordings were made on 22 of the 32 electrodes. The remaining electrodes were used for ground, an online common reference channel (at FCz position, preserved after re-referencing), electrooculogram (EOG), electrocardiogram (ECG), electromyogram (EMG), and mastoid (A1 & A2) recordings. The EEG sampling rate for recording was at 1000 Hz; after being amplified (BrainVision ActiCHamp), data were re-referenced to the contralateral mastoid (A1 & A2) post-recording. The scoring of the sleep data included only eight scalp electrodes (F3, F4, C3, C4, P3, P4, O1, and O2), the EMG, and the EOG. For EEG, EOG, and EMG, low pass filters were set at 35 Hz, high pass filters at 0.3 Hz, and a 60 Hz notch filter was used. Using HUME, a MATLAB toolbox, the raw data were visually scored into Wake, N1, N2, N3, and rapid eye movement sleep (REM) in 30-sec epochs using the American Academy of Sleep Medicine guidelines [72]. The number of minutes from lights out till the first epoch of sleep was estimated as sleep onset latency (SOL) and this same method was used to calculate each stage-dependent latency. Minutes in each stage of sleep (N1, N2, N3, and REM) were calculated. Sleep efficiency (SE) was computed as the sum of the time spent sleeping after lights out divided by the sum of the time spent in bed multiplied by 100.

#### Spectral analyses

Using the Fast Fourier Transformation, the EEG power spectrum was calculated. First, the EEG epochs were bandpass filtered (0.3–35 Hz). Next, any data contaminated by muscle and other artifacts were rejected using a simple out-of-bounds test (with a ± 200 μV threshold). Power spectral density (PSD) was estimated using Welch's method. Absolute band power (μV^2^) in the delta (1–4 Hz) and sigma (12–16 Hz) frequency bands was then obtained by integrating the PSD over the corresponding frequency ranges. Absolute delta and sigma band power were subsequently averaged bilaterally within each sleep stage and subject. We conducted statistical analyses with delta (averaged across F3 and F4 channels) and sigma activity (averaged across C3 and C4 channels) due to previous findings detailing their impact on emotions [37, 73]. To combine spectral measures across NREM stages, delta and sigma values were averaged across N2 and N3 within each participant when usable estimates were available in both stages; when only one stage was available, that value was retained. NREM EEG variables were available for 57 younger and 34 older participants. Descriptive statistics for all sleep architectural variables are presented in Table 2.

**Table 2:** Descriptive statistics of sleep architecture and autonomic variables by age cohort

Sleep variables	Young (M ± SD)	Old (M ± SD)	*P*-value
WASO (min)	6.51 ± 6.76	18.46 ± 13.05	**<.001**
SE	77.36 ± 16.94	54.64 ± 22.18	**<.001**
TST	61.02 ± 22.33	45.29 ± 24.59	**<.001**
SOL(min)	7.68 ± 7.63	9.82 ± 8.06	.166
N1 (min)	4.46 ± 3.46	7.51 ± 6.17	**.002**
N2 (min)	31.11 ± 14.66	28.40 ± 17.97	.397
N3 (min)	19.11 ± 15.06	6.80 ± 9.08	**<.001**
NREM (min)	49.55 ± 18.	35.19 ± 23.61	**<.001**
REM (min) EEG Power (μV^2^)	6.35 ± 8.31	2.58 ± 5.83	**.01**
NREM Delta Power	195.29 ± 89.26	170.16 ± 95.20	.334
NREM Sigma Power	5.52 ± 2.39	5.08 ± 7.91	.721
N2 Delta Power	122.83 ± 71.62	113.38 ± 83.88	.569
N3 Delta Power	261.87 ± 130.99	218.83 ± 133.91	.256
N2 Sigma Power	5.13 ± 2.32	3.58 ± 2.09	.002^*^
N3 Sigma Power HRV During Sleep	5.92 ± 3.14	6.88 ± 15.67	.671
lnHF (NREM)	6.99 ± 0.97	5.39 ± 1.58	**<.001**
lnLF (NREM)	6.38 ± 0.72	5.35 ± 1.38	**<.001**
lnHF (N2)	7.05 ± 0.92	5.37 ± 1.61	**<.001**
lnHF (N3)	6.62 ± 0.67	5.43 ± 1.34	**<.001**
lnLF (N2)	6.92 ± 1.16	5.24 ± 1.54	**<.001**
lnLF (N3)	5.78 ± 0.92	4.69 ± 1.65	.029
Resting HRV			
lnHF (Resting)	6.77 ± 1.05	5.67 ± 1.77	**.001**
lnLF (Resting)	6.57 ± 0.76	5.62 ± 1.49	**<.001**

Note. Values represent mean ± SD. Bold values indicate predictors reaching statistical significance after false discovery rate (FDR) correction. WASO = Wake After Sleep Onset; SE = Sleep Efficiency; TST = Total Sleep Time; SOL = Sleep Onset Latency.

#### Heart rate variability

Electrocardiogram (ECG) data was collected using a modified Lead II Einthoven setup at a sampling rate of 1000 Hz. We used Kubios HRV Analysis Software 2.2 (Biosignal Analysis and Medical Imaging Group, University of Kuopio, Finland) to assess the R-waves series throughout the entire sleep/wake period. Our data processing approach was aligned with the recommendations of the HRV Task Force [57]. We utilized the Kubios software to automatically identify RR peaks, which were then visually inspected and validated by trained technicians. R-peaks that were incorrectly detected were manually modified. Using cubic spline interpolation, missing beats were rectified. The Kubios software’s automated medium filter was used to eliminate artifacts.

The HRV evaluation of the RR series was done with an independent lab tool. Consecutive, artifact-free periods of uninterrupted sleep were chosen during the nap to study RR, HR, and frequency-domain HRV measurements throughout each sleep stage. Each window lasted for three minutes, and there were no stage shifts or movement artifacts during the 1.5 minutes that preceded the window or the full three minutes of the window. The absolute spectral power (ms2) in the LF-HRV (0.04–0.15 Hz; ms2) and the HF-HRV (0.15–0.40 Hz; ms2) frequency bands was computed utilizing an autoregressive model with the order set at 16 [74]. HF-HRV normalized units (HFnu = (HF-HRV [ms2]/ HF-HRV [ms2] + LF-HRV [ms2])^*^100) were derived from these measures as well. HRV outputs were averaged within the five-minute waking interval, N2, N3, and REM sleep periods.

Prior to inferential analysis, the distributions of all HF and LF HRV variables (wake, NREM, N2, and N3) were evaluated separately within younger and older adult cohorts using Shapiro–Wilk tests, skewness and kurtosis statistics, and visual inspection of histograms and Q-Q plots. Positively skew was detected for both HF and LF in their raw form within both age groups (skewness range across variables and groups: 0.60 to 3.20; kurtosis range: −0.15–10.94; Shapiro–Wilk *p* < .01 for all variables except LF_N2 and LF_NREM in younger adults). To adjust for skew, HF and LF power values were natural log-transformed prior to inferential analyses. Following transformation, distributions approximated normality within both age cohorts (|skewness| < 1.0; |kurtosis| < 1.5; all Shapiro–Wilk *p* > .05 within each group). All subsequent analyses involving HF or LF HRV were conducted on natural log-transformed values (lnHF, lnLF). Homogeneity of variance across age cohorts was assessed using Levene's test prior to between-groups comparisons. To evaluate robustness of findings, primary regression models were additionally re-estimated excluding one observation identified as influential via Cook's distance (D > 1). This exclusion of this participant did not significantly impact interpretation. Results from both analytic strategies are provided in the results.

It is common for participants to experience frequent stage transitions during naps, which limits the number of consolidated sleep epochs available for stage dependent HRV analysis. As a result, fewer participants were available for stage dependent HRV analyses (Older N2 = 30, Older N3 = 15, Older REM = 2; Younger N2 = 41, Younger N3 = 34, Younger REM = 16) than who achieved sleep during the nap (Older n = 48; Younger n = 57). Given this methodological limitation of naps, the stage-dependent models included outcomes for NREM sleep only. NREM HRV (Older = 30, Younger = 43) outcomes were derived from N2 and N3 such that values were averaged when both stages were present. If HRV outcomes for only one stage was available, then that value was retained. Models with REM sleep were not feasible due to the low number of both older and younger participants with REM-based HRV outputs during the nap. Degrees of freedom are reported for each analysis to help with interpretation of findings.

### Statistical approach

Hypothesis I: **Age cohort associated with affect ratings**. We hypothesized that older adults would demonstrate relatively greater increases in positive versus negative affect ratings after the nap compared with younger adults. In addition, we expected that participants would exhibit an increase in low vs high arousal affect ratings after the nap. To assess this, positivity ratings, calculated as the difference in positive and negative affect after the nap, were included as dependent outcomes in a repeated measures ANCOVA (RM ANCOVA). Arousal (high vs. low) was included as the within-subject factor and age cohort (younger vs. older) as the between-subject factor. Pre-sleep positivity ratings (low and high arousal) were added as covariates in the model to account for the baseline affect. An interaction between arousal and age was tested. An alpha level of 0.05 was used to determine significance.

Hypothesis II. **Age cohort associated with sleep and autonomic outcomes.** Independent t-tests were used to examine differences in sleep architecture (N2, N3, REM minutes, NREM mins sigma power, delta power, and HRV (lnHF- and lnLF-) in N2, N3, NREM sleep across age cohorts. To control for multiple comparisons, an FDR correction was applied, adjusting the significance threshold to α = .003 (0.05/15). Levene’s tests were used to check normality assumptions were satisfied. If normality was violated, Welch’s correction was applied and degrees of freedom were adjusted accordingly.

**Hypothesis III, IV: Sleep and autonomic outcomes associated with subsequent affective state.** We used multiple linear regressions to assess if autonomic correlates during wake and NREM sleep were associated with post-sleep positivity ratings. We conducted four primary analyses. For wake, lnLF and lnHF indices during a 5-minute period of rest, prior to the nap, and age cohort were included as predictors of both low (1) and high (2) arousal positivity ratings after the nap. For NREM sleep, NREM delta power, sigma power, lnHF lnLF were included as predictors of low (3) and high (4) arousal positivity ratings after the nap. In all models, the sleep neural, autonomic, and age cohort predictors were included and the corresponding pre-sleep positivity rating was entered to account for baseline affect. We report overall model fit and individual predictor coefficients are interpreted. Exploratory regression models examined stage-specific N2 and N3 features separately and are reported in Supplementary Materials Section 1.1 (See Supplemental Figure 2 for the low-arousal positivity rating regression models and Supplemental Figure 3 for the high-arousal positivity rating regression models.). All analyses used listwise deletion and degrees of freedom are reported to assist with interpretation.

Hypothesis V: **Affect associations with subsequent autonomic activity during wake and sleep.** To examine whether affect prior to sleep was associated with autonomic activity, hierarchical linear regression analyses were conducted predicting wake lnHF, wake lnLF, NREM lnHF, and NREM lnLF HRV from pre sleep positivity ratings. Separate models were conducted for each HRV outcome. In each model, age cohort and the relevant pre sleep positivity rating was entered at Step 1. At Step 2, the interaction between age and pre sleep was added to test whether associations between pre sleep affect and HRV differed across younger and older adults. If significant interactions emerged, they were probed using follow up simple slopes analyses within each age cohort. An alpha level of .05 was used to determine significance. Exploratory analyses probed if associations differed during N2 and N3 sleep. Findings are reported in the Supplementary Materials Section 1.2. All analyses were performed using SPSS (IBM SPSS Statistics version 28.0).

## Results

### Arousal dependent positivity ratings post nap differs by age

**Figure 2:**
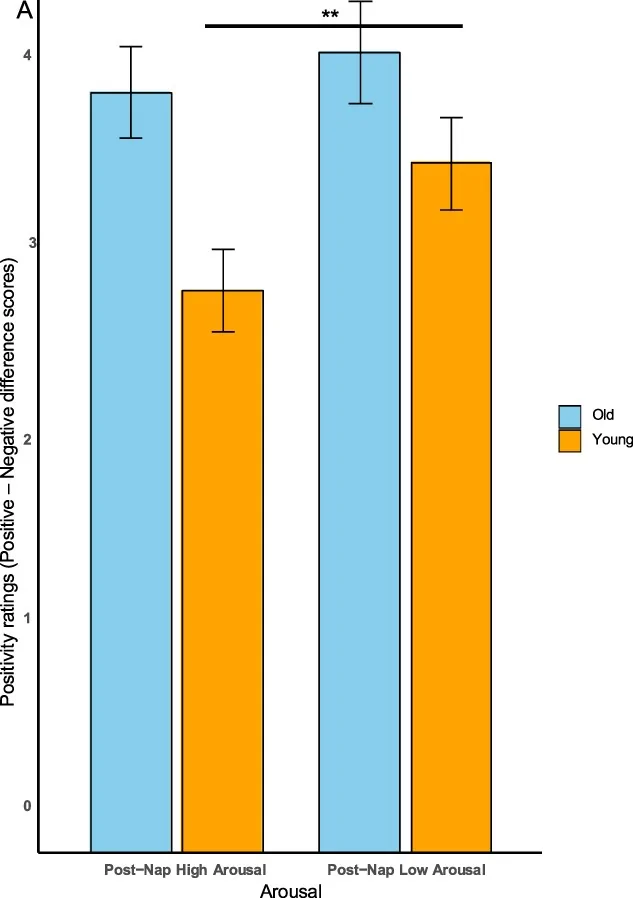
(A) Mean post nap positivity ratings for high and low arousal emotions in younger and older adults. Positivity ratings represent the difference between positive and negative affect ratings within each arousal level (low vs. high), with higher values indicating greater positive than negative affect endorsement. Bars depict mean ratings for younger (orange) and older (blue) adults in each condition. Younger adults exhibited significantly greater positivity ratings for low arousal compared to high arousal emotions following the nap, whereas older adults did not show a significant difference between arousal conditions. Error bars represent ± SE. *p* < .01.

After controlling for pre-nap positivity ratings, post nap positivity ratings were dependent on age cohort (Age × Arousal interaction: (F [1,95] = 7.143, *p* = .009, ηp^2^ = 0.070) (Younger n = 55, Older n = 44)). Young adults expressed stronger low arousal positivity ratings (Mean ± SE = 3.616 ± .165) compared to high arousal (Mean ± SE = 2.970 ± 0.141, *p* <0.001) after the nap, whereas no difference emerged between high (Mean ± SE = 3.34 ± .159) and low arousal (Mean ± SE = 3.412 ± 0.186) positivity ratings for older adults (*p* = .632). Refer Figure 2.

### Parasympathetically-mediated autonomic activity during wake and sleep is dampened with age.

There were significant age dependent differences in lnHF (Mean Diff ± SE = 1.11 ± 0.33), t(55.39) = 3.37, *p* = .001, and lnLF (Mean Diff ± SE = 0.96 ± 0.27), t(51.15) = 3.55, *p* < .001) during wake, with a dampening of both lnHF and lnLF in older compared to younger adults.

Similarly, dampening of lnHF and lnLF during NREM sleep was observed in older relative to younger adults. lnHF during NREM sleep was significantly higher in young adults compared to older adults (Mean Diff ± SE = 1.61 ± 0.33, t(44.23) = 4.93, *p* < .001), as was lnLF during NREM sleep (Mean Diff ± SE = 1.03 ± 0.28, t(40.21) = 3.73, *p* < .001). lnHF during N2 sleep was significantly higher in young adults compared to older adults (Mean Diff ± SE = 1.68 ± 0.33, t(42.69) = 5.10, *p* < .001), as was lnHF during N3 sleep (Mean Diff ± SE = 1.67 ± 0.45, t(21.29) = 3.74, *p* = .001). lnLF during N2 (Mean Diff ± SE = 1.19 ± 0.27, t(39.81) = 4.44, *p* < .001) and N3 sleep (Mean Diff ± SE = 1.09 ± 0.46, t(17.93) = 2.38, *p* = .029) was also higher in younger when compared to older adults; however, the lnLF difference during N3 did not meet the FDR-corrected significance threshold. All wake and sleep-related outcomes are included in Table 2.

### Autonomic activity is associated with subsequent affective experience

#### Waking autonomic activity is linked to post-nap emotions

Resting autonomic activity and age cohort explained significant variance in post-nap positivity for both low-arousal, R^2^ = 0.535, F [4,68] = 19.531, *p* < .001, and high-arousal stimuli, R^2^ = 0.638, F [4,68] = 30.001, *p* < .001. Higher lnHF_wake was associated with lower post-nap positivity for both low- (β = −0.317, *p* = .038) and high-arousal (β = −0.264, *p* = .049), whereas higher lnLF_wake was associated with greater positivity for both low- (β = 0.354, *p* = .021) and high-arousal (β = 0.292, *p* = .031). Age cohort was associated with high-arousal positivity (β = 0.171, *p* = .045) but not low-arousal positivity (*p* = .724). Baseline positivity ratings were the strongest predictor of post-nap affect in each model (low-arousal ΔR^2^ = 0.491, β = 0.725; high-arousal ΔR^2^ = 0.458, β = 0.710; both *p* < .001). Full standardized coefficients are reported in Supplementary Table S1.

#### Autonomic activity during NREM sleep is linked to post-nap positivity ratings

**Figure 3:**
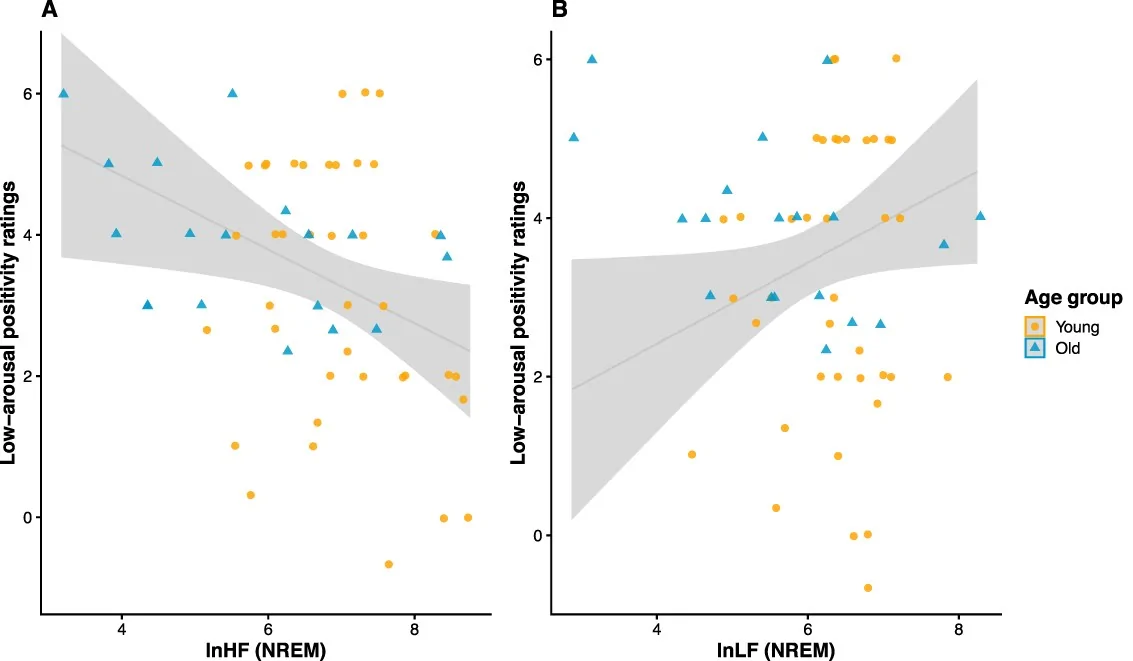
(A) Associations between autonomic activity during NREM sleep and post-sleep low-arousal positivity ratings. lnHF during NREM sleep. Higher lnHF during NREM was associated with lower post-nap low-arousal positivity ratings. (**3B)** lnLF during stage NREM sleep. Higher lnLF during NREM was associated with higher post-nap low-arousal positivity ratings. Lines represent marginal predictions from linear regression models relating sleep physiological variables (lnHF and lnLF) to post-nap low-arousal positivity ratings while holding other covariates constant at their mean values. Shaded areas indicate 95% confidence intervals. Points represent individual participants and are color-coded by age group (young adults: Orange circles; older adults: Blue triangles).

**Figure 4:**
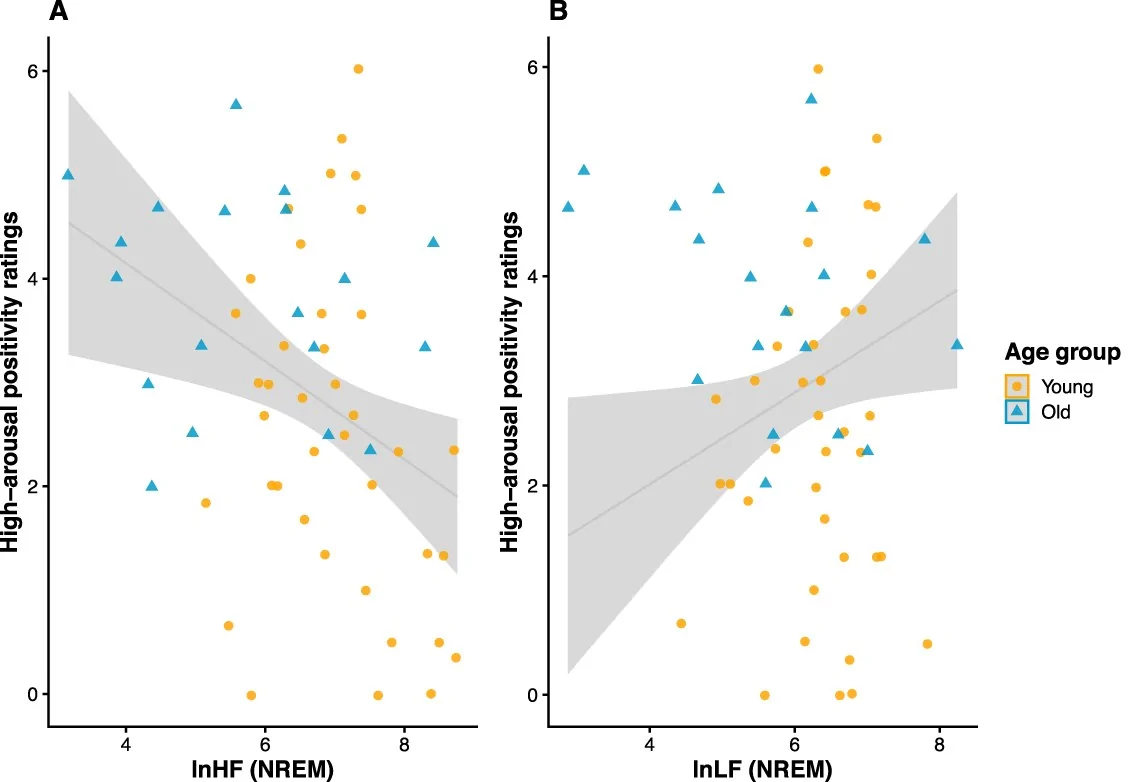
Associations between sleep physiological activity during NREM sleep and post-sleep high-arousal positivity ratings. Higher lnHF during NREM was associated with lower post-nap high-arousal positivity ratings. Lines represent marginal predictions from linear regression models relating sleep physiological variables to post-nap high-arousal positivity ratings while holding other covariates constant at their mean values. Shaded areas indicate 95% confidence intervals. Points represent individual participants and are colored by age group (young adults, orange circles; older adults, blue triangles).

NREM autonomic activity, NREM sleep indices, and age cohort also significantly predicted low, R^2^ = 0.516, F [7,50] = 7.605, *p* < .001 and high R^2^ = .647, F [7,50] = 13.077, *p* < .001 arousal positivity. Higher lnHF_NREM was associated with lower post-nap positivity for both low- (β = −0.413, *p* = .017) and high-arousal (β = −0.397, *p* = .007) positivity ratings, whereas higher lnLF_NREM was associated with greater positivity for both low- (β = 0.321, *p* = .044) and high-arousal ratings (β = 0.291, *p* = .033; Figures 3 and 4). No other NREM sleep or age predictor reached significance (all ps ≥ 0.210). Baseline ratings were also robustly associated with both low- (ΔR^2^ = 0.365, F [1,50] = 37.688) and high-arousal positivity (ΔR^2^ = 0.436, F [1,50] = 61.757; both *p* < .001). Full standardized coefficients are reported in Table 3. To evaluate robustness of findings, primary regression models were additionally re-estimated excluding one observation identified as influential via Cook's distance (D > 1). The pattern of effects was largely preserved: lnHF remained a significant predictor of both high-arousal and low-arousal post-nap positivity ratings in the case-excluded models, while lnLF showed associations in the same direction that approached but did not reach significance (ps = 0.051–0.069). Stage-specific associations are presented in Supplemental Figures 2 and 3, and the corresponding N2 and N3 regression models are reported in Supplemental Tables 2 and 3, respectively.

**Table 3:** Multiple regression models predicting post nap high and low arousal positivity ratings from corresponding pre sleep positivity ratings, NREM autonomic variables, NREM neural variables, NREM duration, and age group

Model fit	Variable	β	t	*P-*value	Partial r
**Panel A. Post nap high arousal positivity**					
R^2^ = 0.647, adjusted R^2^ = 0.597; F (7, 50) = 13.077, *p* < .001	lnHF NREM	−0.397	−2.790	**.007**	−0.367
	lnLF NREM	0.291	2.194	**.033**	0.296
	NREM minutes	0.007	0.070	.945	0.010
	NREM delta	0.052	0.526	.601	0.074
	NREM sigma	−0.120	−1.269	.210	−0.177
	Age group	0.028	0.274	.785	0.039
	Pre sleep high arousal positivity	0.721	7.859	**<.001**	0.743
**Panel B. Post nap low arousal positivity**					
R^2^ = 0.516, adjusted R^2^ = 0.448; F (7, 50) = 7.605, *p* < .001	lnHF NREM	−0.413	−2.463	**.017**	−0.329
	lnLF NREM	0.321	2.068	**.044**	0.281
	NREM minutes	0.035	0.309	.758	0.044
	NREM delta	0.086	0.744	.460	0.105
	NREM sigma	−0.084	−0.763	.449	−0.107
	Age group	−0.072	−0.621	.537	−0.088
	Pre sleep low arousal positivity	0.665	6.139	**<.001**	0.656

**Note**. N = 58. Panel A predicts post nap high arousal positivity; Panel B predicts post nap low arousal positivity. Each model included the corresponding pre sleep positivity rating, lnHF NREM, lnLF NREM, NREM minutes, NREM delta power, NREM sigma power, and age group entered simultaneously. β = standardized beta coefficient. Partial r = partial correlation coefficient. lnHF = natural log transformed high frequency heart rate variability; lnLF = natural log transformed low frequency heart rate variability; NREM = non rapid eye movement sleep. Bold-type text indicates significant differences at the apriori determined alpha level for each analysis.

### Associations between pre nap positivity ratings and subsequent autonomic activity

Positivity ratings were not associated with wake lnHF or lnLF (p’s 0.46–0.95). No age group × positivity ratings interactions emerged (all ps ≥ 0.107). Similarly, positivity ratings were not meaningfully associated with subsequent NREM lnHF or lnLF (p’s 0.10–0.92), and no significant age × positivity interactions were statistically meaningful (all ps ≥ 0.060).

## Discussion

The current study examined bidirectional associations between affect, arousal, sleep architecture, and cardiac autonomic activity across a daytime nap in healthy younger and older adults. Findings indicate that autonomic activity during a resting wake and sleep interval are associated with affect and arousal in young and older adults in nuanced ways that may help shed light on affective regulation.

Two theories are central to the theoretical underpinnings of positivity shifts with aging: Socioemotional Selectivity Theory (SST) and Strength and Vulnerability Integration (SAVI). According to the SST, the positive emotional shift in behavior with age can be due to the awareness that people develop of their limited time in their life span, directing them to prioritize emotionally meaningful and positively-valenced experiences [7, 21, 22]. According to SAVI, individuals attain knowledge and experience with aging that can help them to downregulate negative affect, and aid in the upregulation of positive affect [75]. Although older adults demonstrated numerically higher positivity ratings than younger adults both before and after sleep, we did not observe a statistically significant effect of age, which place our findings in minor conflict with both SAVI and SST tenets. Importantly, one aim of the present study was to examine whether positivity ratings vary across sleep and as a function of both age and emotional arousal. Indeed, the primary age-related difference that emerged in the dataset was in terms of arousal: younger adults showed clear differentiation between high- and low-arousal positivity ratings following the nap, whereas older adults did not. One possible explanation is that older adults exhibit stable rates of positive and negative emotions, whereas younger adults experience greater affective fluctuations[16, 76, 77]. Alternatively, aging may be associated with reduced differentiation in emotional arousal, which has been previously suggested [78]. As we did not experimentally manipulate emotional states, future work combining affective modulation paradigms with sleep monitoring is needed to disentangle the mechanisms underlying these age-related arousal effects.

It remains unclear whether age-related positivity reflects unconscious strategies that older adults implement or an accompanying change in neurobiology. Prior evidence suggested a bidirectional relationship between parasympathetic activity and positive affect [66, 79, 80]. Here, we examined both how pre-sleep affective ratings impacted autonomic activity during rest, and sleep, as well as how rest and sleep autonomic activity impacted subsequent affect. Explicitly, both LF during resting wakefulness and NREM sleep were positively associated with stronger positivity ratings after the nap for low arousal emotions, and NREM HF during rest and NREM sleep with dampened positivity ratings after the nap for both arousal categories. These relationships persisted after accounting for pre-sleep emotions. The autonomic-affective patterns presented here are aligned with prior evidence that high-arousal positive emotions are accompanied by greater variability in the LF/HF ratio during wakefulness [81]. Alternatively, we found little evidence that pre-sleep positive affect or arousal was reliably associated with autonomic activity during either NREM sleep or waking rest, which calls into question just how much state-dependent fluctuations in affective experiences are hard coded in subsequent autonomic physiology. Instead, our findings extend existing research to acknowledge that autonomic contexts during wake and sleep likely provide a milieu for which affective experiences can be projected, and that autonomic-affective associations are shaped in consistent ways across the lifespan.

Our findings conflict with previous work indicating that HF HRV is positively associated with calm, low-arousal affective states (e.g. contentment, safeness) [82]. Though we found inverse relationships between sleep and wake HF-HRV and low arousal positive emotions both before and after the sleep interval. However, our study protocol did not focus on emotional states explicitly tied to safety. Other work has suggested that the relationship between HF HRV and low arousal emotional states may not be linear [82], yet we did not find evidence for a non-linear relationship between HF HRV and emotional states during sleep or wake. Alternatively, we found positive associations between LF HRV during wake and positivity ratings after a sleep experience. A recent review noted that associations between HRV and related outcomes likely depend on the HRV metric, the affective experience measured, and whether affect is assessed at the state- versus trait-level [83]. Therefore, the fact that we examined affective states and autonomic fluctuations peri-sleep may have greatly influenced the associations reported. More studies are needed to disentangle how various HRV metrics map onto types of affect, and how these patterns shift across arousal states, contexts, and aging. Theoretical models considering sleep and arousal dynamics would be helpful in guiding future empirical work.

Importantly, the present findings do not indicate that NREM sleep broadly predicted affective outcomes. Rather, the significant effects were specific to autonomic activity measured during NREM, with HRV indices associated with post-nap positivity whereas NREM duration, delta power, and sigma power were not significant predictors. These results suggest that the affect may be more closely tied to autonomic regulation during NREM sleep than to NREM sleep quantity or canonical oscillatory features. Due to the combination of short daytime nap duration and the reduction of slow-wave sleep in older adulthood limited the number of older participants with usable N3-based measures. Models adjusting for NREM EEG spectral covariates were estimated in n = 19 older adults, and age-stratified inferences from these models should be interpreted with caution and warrant replication in protocols using longer sleep opportunities.

Autonomic dampening was observed within the older cohort during both wake and sleep. Previous studies examining age-related differences in sleep-associated ANS activity during both nighttime [84, 85] and daytime [64, 65] sleep have reported similar findings. Studies have suggested that napping can be a restorative strategy for regulating emotions and promoting well-being [86–88]. It has been shown that the propensity of napping increases with age [89] and that napping can impact both adaptive and maladaptive psychosocial and health outcomes [90–92]. Our findings suggest that age-related reductions in parasympathetic activity during naps may attenuate the positivity effect—a hallmark of adaptive aging. We contend that this attenuation should be interpreted alongside mixed evidence that habitual napping in older adults has been associated with heightened risk for mood and arousal disorders [93, 94].

Lastly, sympathetic drive, typically associated with negative emotional states, is markedly reduced directly before and during NREM sleep, and parasympathetic dominance peaks during NREM sleep [67, 68, 95, 96]. Thus, we purport that reduced LF- and HF-HRV in NREM sleep states may indicate a specific downregulation of autonomic tone [84, 85, 97, 98] that, alongside physiological arousal mechanisms during wake, serve to calibrate post-sleep affective states consistently across the lifespan. Importantly, given our cohort design, we cannot assume causality. Further, it is possible that autonomic activity and emotional states are related through other psychophysiological phenomena that change together with aging. Future studies should leverage affective, autonomic, and/or sleep interventions to determine causal relationships. Prospective designs and longitudinal cohorts can also help illuminate age-dependencies.

### Limitations

Emotions were assessed twice (~15 minutes pre and post nap), but the post nap assessment was limited to a single time point, preventing us from characterizing the course of affective change after sleep. It is known that NREM sleep, especially N3, and related sleep physiological features are associated with sleep inertia [99], and grogginess upon awakening can increase negative affect, including feelings of irritability and frustration [100–102]. However, relief from sleep pressure may attenuate some of these initial sleep and emotion relationships [25]. The timing of our affect ratings may have allowed sleep inertia to play a role in our findings [99]. Importantly, affective ratings were obtained from both older and younger adults, at similar delays following awakening, suggesting that any potential influence of sleep inertia is comparable across age groups.

The present study examined state-level assessments of both affect and autonomic activity. Our metrics did not capture chronic affective dispositions or trait-level autonomic tendencies that may emerge across longer time scales and varied contexts. Future research incorporating repeated assessments across multiple days, weeks, or contexts could help illuminate stability and reliability of the short-term associations demonstrated here.

A daytime nap protocol was utilized in the current investigation. Daytime naps are an important aspect of sleep within 24 hours for younger and older adults [103]. With aging, daytime napping may also become more prominent [104]. However, given the time constraints of a 90-minute daytime nap opportunity, many of our participants in the study did not receive sufficient amounts of continuous sleep to conduct stage-dependent HRV analyses. These findings should be interpreted cautiously given the uneven distribution of younger and older adults and incomplete sleep-stage data, particularly for N3. Future studies can assess sleep physiological activity during a nighttime sleep interval to examine if similar sleep-dependent relationships emerge.

We leveraged a cross-sectional design to examine age, yet prospective methods that examine sleep and affective changes over time are warranted, especially given longitudinal associations linking aging to positive and negative affect are nuanced [105]. Lastly, the mechanistic causes of HRV are debated [56, 57, 106]. As such, interpretation of the contributing physiological factors to our results remains an open question. This highlights the need for a deeper mechanistic understanding of HF and LF-HRV to better comprehend the observed links between sleep and autonomic contributions to affective well-being.

## Conclusion

Our findings point to waking and NREM-linked autonomic activity as a pathway through which affective experience may be calibrated across the lifespan. This work adds to a growing body of literature emphasizing the importance of arousal in determining age-dependent associations between sleep and emotions and further indicate a nuanced relationship between wake and sleep autonomic activity as important signals molding daily affective experience. Future research should build on this approach with overnight sleep studies and experimental manipulations to test whether enhancing specific features of sleep and/or autonomic activity can be used to support emotional well-being.

## Supplementary Material

Supplemental_Materials_clean_zpag083(2)
