# Supplementary material for: Sleep dependent autonomic correlates of affect and arousal in younger and older adults
Source: Sleep Adv. 2026 Jul 28;7(3):zpag083. doi: 10.1093/sleepadvances/zpag083 (PMC13537464; doi:10.1093/sleepadvances/zpag083)
Supplement: Supplementary file 1 [file supplemental_materials_clean_zpag083(2).docx]

**Supplemental Materials:**

**Sleep Dependent Autonomic Correlates of Affect and Arousal in Younger and Older Adults**

Authors: Anjana Subramoniam^1^, Pin-Chun Chen^2^, Negin Sattari^3^, Sara C. Mednick^3^, Lauren N. Whitehurst^4,5,6^

1. Department of Psychiatry, College of Medicine, University of Pittsburgh, USA
2. Department of Psychology, Royal Holloway, University of London, Egham, TW20 0EX, United Kingdom
3. Cognitive Science School of Social Sciences, University of California, Irvine, USA
4. Department of Psychology, Yale University, USA
5. Department of Black Studies, Yale University, USA
6. Wu Tsai Institute, Yale University, USA

Corresponding author:

Lauren N. Whitehurst, PhD

Yale University

Email: [lauren.whitehurst@yale.edu](mailto:lauren.whitehurst@yale.edu) Telephone: (203)-432-1448

**Supplemental Fig1.** Mean ratings (± SE) across age groups and conditions before and after the nap. Bars depict mean ratings for Younger (orange) and Older (blue) adults in each condition: High Arousal Positive, Low Arousal Positive, High Arousal Negative, and Low Arousal Negative. Data are shown separately for the pre-sleep and post-sleep assessments.

**1.1. Autonomic activity during N2 and N3 sleep is linked to post-nap positivity ratings:**

For low-arousal positivity, the N2 model accounted for a significant proportion of variance in post-nap low-arousal positivity ratings (R² = 0.557, F(7, 48) = 8.607, p < 0.001). However, no individual N2 sleep or autonomic predictor reached significance; lnHF during N2 showed only a non-significant trend toward reduced positivity ratings (β = −0.289, p = 0.078), and lnLF was not associated with positivity ratings (β = 0.163, p = 0.323) (all other ps ≥ 0.085). For N3 sleep, our set of predictors explained a significant proportion of variance in post-nap low-arousal positivity ratings (R² = 0.635, F(7, 29) = 7.206, p < 0.001). Higher lnHF during N3 sleep was associated with less positivity after the nap (β = −0.412, p = 0.033) (Refer Figure 3B), whereas lnLF showed the opposite association (β = 0.369, p = 0.038). No other predictors were significant (all ps ≥ 0.150). Refer Supplementary Fig 1.

For high-arousal positivity, the N2 model accounted for a significant proportion of variance in post-nap high-arousal positivity ratings, R² = 0.660, F(7, 48) = 13.327, p < 0.001 (analytic n = 56). lnHF during N2 was inversely associated with high-arousal positivity (β = −0.314, p = 0.030), whereas lnLF during N2 was not significant (β = 0.233, p = 0.111). N2 sigma power showed a non-significant inverse trend (β = −0.194, p = 0.069); no other predictors reached significance (all ps ≥ 0.181). For N3 sleep, the model also accounted for a significant proportion of variance in post-nap high-arousal positivity, R² = 0.674, F(7, 29) = 8.564, p < 0.001 (analytic n = 37). lnHF during N3 was significantly associated with reduced post-nap high-arousal positivity (β = −.0409, p = 0.023), whereas lnLF during N3 was not significant (β = 0.182, p = 0.266). No other predictors were significant (all ps ≥ .446). See Supplementary Figure S2.

**1.2** **Associations between pre-nap positivity ratings and autonomic activity during N2 and N3 sleep**

***For low-arousal positivity***,

For N2, the main-effects models including low positive bias and age group were significant for both LF-HRV, R² = 0.320, F(2, 65) = 15.26, p < 0.001, and HF-HRV, R² = 0.333, F(2, 65) = 16.24, p < 0.001. However, the age group × low positive bias interaction was not significant for LF-HRV, ΔR² = 0.028, p = 0.102, or HF-HRV, ΔR² = 0.004, p = 0.532. For N3, the main-effects models were significant for both LF-HRV, R² = .287, F(2, 45) = 9.05, p < 0.001, and HF-HRV, R² = 0.401, F(2, 45) = 15.08, p < 0.001. The age group × low positive bias interaction was significant for N3 LF-HRV, ΔR² = 0.092, ΔF(1, 44) = 6.53, p = .014, but not for N3 HF-HRV, ΔR² = 0.016, p = 0.284.

***For high-arousal positivity***,

For N2, the main-effects models including high positive bias and age group were significant for both LF-HRV, R² = 0.260, F(2, 65) = 11.43, p < 0.001, and HF-HRV, R² = 0.285, F(2, 65) = 12.93, p < 0.001. However, the age group × high positive bias interaction was not significant for LF-HRV, ΔR² = 0.031, p = 0.097, or HF-HRV, ΔR² = 0.001, p = 0.738. For N3, the main-effects models were significant for both LF-HRV, R² = 0.174, F(2, 45) = 4.73, p = 0.014, and HF-HRV, R² = 0.287, F(2, 45) = 9.06, p < 0.001. The age group × high positive bias interaction was significant for N3 LF-HRV, ΔR² = 0.094, ΔF(1, 44) = 5.63, p = 0.022, but not for N3 HF-HRV, ΔR² = 0.018, p = 0.297.

**Supplemental Figure 2**

**Associations between autonomic activity during N3 sleep and post-sleep low-arousal positivity ratings.**

Marginal prediction plots derived from linear regression models illustrating the associations between (A) high-frequency heart rate variability (lnHF) during N3 sleep, and (B low-frequency heart rate variability (lnLF) during N3 sleep with post-sleep low-arousal positivity ratings. Regression lines represent model-estimated predicted values across the observed range of each physiological predictor while holding other covariates in the model constant. Shaded regions indicate 95% confidence intervals around the predicted values. Points represent individual participants and are color-coded by age group (young adults: orange circles; older adults: blue triangles).

**Supplemental Figure 3**

**Associations between sleep physiological activity during NREM sleep and post-sleep high-arousal positivity ratings** :
Marginal prediction plots derived from linear regression models illustrating the associations between (A) high-frequency heart rate variability (lnHF) during N2 sleep, (B) low-frequency heart rate variability (lnLF) during N2 sleep with post-sleep high-arousal positivity ratings. Regression lines represent model-estimated predicted values across the observed range of each predictor while holding other variables in the model constant. Shaded regions indicate 95% confidence intervals around the predicted values. Points represent individual participants and are colored by age group (young adults, orange circles; older adults, blue triangles)

**Supplemental Table 1**. Multiple regression models predicting post nap positivity ratings from pre sleep positivity ratings, wake HRV, and age

| Model | Model fit | Variable | β | t | p | | Partial | |
| --- | --- | --- | --- | --- | --- | --- | --- | --- |
|  | **Panel A. Post nap high arousal positivity** | | | | | | |  |
| Model 1 | R² = .638, F(4, 68) = 30.001, p < .001 | Pre sleep high arousal positivity | 0.710 | 9.282 | | **< .001** | | 0.748 |
|  |  | lnLF_wake | 0.292 | 2.201 | | **0.031** | | 0.258 |
|  |  | lnHF_wake | −0.264 | -2.002 | | **0.049** | | -0.236 |
|  |  | Age | 0.171 | 2.040 | | **0.045** | | 0.240 |
|  | **Panel B. Post nap low arousal positivity** | | | | |  | |  |
| Model 1 | R² = .535, F(4, 68) = 19.531, p < .001 | Pre sleep low arousal positivity | 0.725 | 8.467 | | **< .001** | | 0.716 |
|  |  | lnLF_wake | 0.354 | 2.368 | | **0.021** | | 0.276 |
|  |  | lnHF_wake | -0.317 | -2.115 | | **0.038** | | -0.248 |
|  |  | Age | -0.033 | -0.355 | | 0.724 | | -0.043 |

***Note.*** Both models included pre sleep positivity of the corresponding arousal condition, lnLF_wake, lnHF_wake, and age group. β = standardized beta coefficient. Partial = partial correlation coefficient. Bold values indicate predictors reaching statistical significance.

**Supplemental Table 2. Hierarchical regression models predicting post nap positivity ratings from pre sleep positivity and N2 sleep variables**

| Model | Model fit | Variable | β | t | p | Partial r |
| --- | --- | --- | --- | --- | --- | --- |
| **Panel A. Post-nap high-arousal positivity** | | | | | | |
| Model 2 | R² = 0.660, F(7, 48) = 13.327, p < 0.001 | lnHF N2 | −0.314 | −2.231 | **0.030** | −0.307 |
|  |  | lnLF N2 | 0.233 | 1.625 | 0.111 | 0.228 |
|  |  | S2 minutes | 0.008 | 0.090 | 0.929 | 0.013 |
|  |  | S2 delta | 0.135 | 1.358 | 0.181 | 0.192 |
|  |  | S2 sigma | −0.194 | −1.862 | 0.069 | −0.260 |
|  |  | Age | −0.005 | −0.045 | 0.964 | −0.006 |
|  |  | Pre-sleep high-arousal positivity | 0.720 | 7.736 | **< 0.001** | 0.745 |
| **Panel B. Post-nap low-arousal positivity** | | | | | | |
| Model | R² = 0.557, F(7, 48) = 8.607, p < 0.001 | lnHF N2 | −0.289 | −1.800 | 0.078 | −0.251 |
|  |  | lnLF N2 | 0.163 | 0.999 | 0.323 | 0.143 |
|  |  | S2 minutes | 0.138 | 1.311 | 0.196 | 0.186 |
|  |  | S2 delta | 0.199 | 1.757 | 0.085 | 0.246 |
|  |  | S2 sigma | −0.209 | −1.736 | 0.089 | −0.243 |
|  |  | Age | −0.154 | −1.315 | 0.195 | −0.186 |
|  |  | Pre-sleep low-arousal positivity | 0.675 | 6.364 | **< 0.001** | 0.677 |

*Note.* N = 56. Panel A predicts post-nap high-arousal positivity; Panel B predicts post-nap low-arousal positivity. In both panels, lnHF N2, lnLF N2, S2 minutes, S2 delta power, S2 sigma power, and age cohort were included and the pre-sleep positivity measure was utilized to control for baseline affect. β = standardized beta coefficient. Partial r = partial correlation coefficient. Partial r = partial correlation coefficient. Bold values indicate predictors reaching statistical significance at p < .05.

**Supplemental Table 3. Regression models predicting post nap positivity from pre sleep positivity and N3 sleep variables**

| Model | Model fit | Variable | β | t | p | Partial r |
| --- | --- | --- | --- | --- | --- | --- |
| **Panel A. Post-nap high-arousal positivity** | | | | | | |
| Model | R² = 0.674, F(7, 29) = 8.564, p < 0.001 | lnHF N3 | −0.409 | −2.400 | **0.023** | −0.407 |
|  |  | lnLF N3 | 0.182 | 1.135 | 0.266 | 0.206 |
|  |  | S3 minutes | 0.092 | 0.761 | 0.453 | 0.140 |
|  |  | S3 delta | −0.061 | −0.498 | 0.622 | −0.092 |
|  |  | S3 sigma | −0.096 | −0.773 | 0.446 | −0.142 |
|  |  | Age | 0.011 | 0.086 | 0.932 | 0.016 |
|  |  | Pre-sleep high-arousal positivity | 0.699 | 6.454 | **< 0.001** | 0.768 |
| **Panel B. Post-nap low-arousal positivity** | | | | | | |
| Model | R² = 0.635, F(7, 29) = 7.206, p < 0.001 | lnHF N3 | −0.412 | −2.238 | **0.033** | −0.384 |
|  |  | lnLF N3 | 0.369 | 2.171 | **0.038** | 0.374 |
|  |  | S3 minutes | −0.089 | −0.695 | 0.493 | −0.128 |
|  |  | S3 delta | 0.064 | 0.490 | 0.628 | 0.091 |
|  |  | S3 sigma | −0.195 | −1.480 | 0.150 | −0.265 |
|  |  | Age | −0.083 | −0.603 | 0.551 | −0.111 |
|  |  | Pre-sleep low-arousal positivity | 0.697 | 5.817 | **< 0.001** | 0.734 |

*Note.* N = 37. Panel A predicts post-nap high-arousal positivity; Panel B predicts post-nap low-arousal positivity. In both panels, lnHF N3, lnLF N3, S3 minutes, S3 delta power, S3 sigma power, and age cohort were included and the pre-sleep positivity measure was utilized to control for baseline affect. β = standardized beta coefficient. Partial r = partial correlation coefficient.
